# Predictors for outcome among cardiac arrest patients: the importance of initial cardiac arrest rhythm versus time to return of spontaneous circulation, a retrospective cohort study

**DOI:** 10.1186/s12873-015-0028-3

**Published:** 2015-02-04

**Authors:** Ida Wibrandt, Kristine Norsted, Henrik Schmidt, Jens Schierbeck

**Affiliations:** Department of Anesthesiology and Intensive Care, Odense University Hospital, Odense, Denmark

## Abstract

**Background:**

In the past decade, early treatment of cardiac arrest (CA) victims has been improved in several ways, leading to more optimistic over all prognoses. However, the global survival rate after out-of-hospital CA (OHCA) is still not more than 5-10%. With a better knowledge of the predictors for outcome among CA patients, we can improve the management of CA, in order to strengthen the leads in the chain of survival.

**Methods:**

A retrospective cohort study including 172 CA patients admitted to the intensive care unit (ICU) in Odense University Hospital (OUH) in a three-year period was conducted. We determined the 90-day mortality and neurological outcome at discharge for CA patients treated with therapeutic hypothermia (TH), in regard to determine the importance of the predictors for mortality and neurological outcome, with emphasize on combining initial rhythm and time to return of spontaneous circulation (ROSC).

**Results:**

The overall mortality was 44% and a favorable neurological outcome was seen among 52%. Strong predictors for survival and favorable neurological outcome were ventricular tachycardia/ventricular fibrillation (VT/VF) as initial rhythm, cardiac etiology and time to ROSC < 20 minutes. Age < 60 years was a predictor for survival only. Patients with the combination of VT/VF and ROSC < 20 minutes had undeniably the best chance of both survival and a favorable neurological outcome.

**Conclusions:**

We found significant predictors for both survival and neurological outcome, in which an initial rhythm of VT/VF and a cardiac etiology were the strongest.

## Background

Cardiac arrest (CA) victims have a high risk of death or poor neurologic function. The survival rate of out-of-hospital CA (OHCA) is reported to be 5-10% [1,2]. However, survival rates can reach 20-40% in witnessed cases of OHCA with VF as initial rhythm in communities where the chain of survival is strong [3,4]. It is known that the most common initial rhythm is ventricular fibrillation (VF), with a frequency of 60-80% of all CA. Because VF is a shockable rhythm, the chance of survival is up to 10 times higher compared with the non-VT/VF rhythms [5]. In Denmark, there is approximately 3500 OHCA every year and the 30-day survival rate was 10.1% in 2011. The median age for OHCA patients in Denmark are 70 and 65% of the OHCA patients are males. Strong predictors for 30-day survival are reported to be bystander cardiopulmonary resuscitation (CPR) before the arrival of the ambulance, early defibrillation, short time to return of spontaneous circulation (ROSC) and VT/VF as initial rhythm [4,6].

Therapeutic hypothermia (TH) may be beneficial in treatment of patients achieving ROSC after CA [7-9]. The background for the current recommendations regarding TH is based on two randomized controlled trials (RCT) published in 2002 [8,9], which demonstrated that TH increases the rate of neurological recovery and reduces the mortality after CA. Based on the two articles, the Advanced Life Support (ALS) Task Force of the International Liaison Committee on Resuscitation (ILCOR) in 2003 recommended TH for CA survivals [10] and the treatment was, and still is, included in the European Resuscitation Council Guidelines for resuscitation [11].

A large amount of studies have investigated different outcome measures among CA patients treated with TH and there are many reports on predictors for outcome. Less is known about the importance of these predictors against each other.

The aim of this retrospective analysis was to determine the 90-day mortality and neurological outcome at discharge for CA patients treated with TH, in regard to determine the importance of the predictors for mortality and neurological outcome, with emphasize on combining initial rhythm and time to ROSC.

## Methods

### Study design and patient population

We conducted a retrospective cohort study, where we studied medical records of CA patients admitted to one of the two ICUs in Odense University Hospital (OUH) in Denmark between January 1st 2008 and December 31st 2010. The two ICUs followed the same protocol regarding the management of CA patients.

The criteria for inclusion were:

1. Adults with an age ≥ 18 years, suffering from either in-hospital CA (IHCA) or OHCA with ROSC, regardless of initial rhythm or etiology. We allowed the CA to be both witnessed and un-witnessed.
2. Treatment with TH.

### Data collection

Data was found in the internal database for medical journals. Permission to use the database was given by the management of the Department of Anesthesiology and Intensive Care, OUH. The Regional Committees on Health Research Ethics for Southern Denmark ruled that no formal ethics approval was required in this case.

The following baseline characteristics were registered from the medical records; age, gender, number of days alive, mortality at discharge, 30 and 90 days, location of CA (IHCA or OHCA), initial rhythm, time from collapse to ROSC (<20 min. or ≥ 20 min.), etiology and discharge destination (home/rehabilitation facility or nursing home/death). We chose to register time to ROSC as < or ≥ 20 minutes, to make the results comparable with other studies in the field [12-16]. We registered the first recorded rhythm during CA as the initial rhythm. Favorable neurological outcome was defined as a Cerebral Performance Category (CPC) of 1–2 (discharge to home or rehabilitation facility) and unfavorable neurological outcome was defined as CPC 3–5 (discharge to nursing home or death) [17].

### Outcome assessment

The primary outcomes were overall mortality at 90 days from the CA and neurological outcome at discharge from hospital. We compared the initial rhythm and the time to ROSC as predictors for mortality and neurological outcome. Secondly, we investigated other predictors for outcome.

### Cooling

Cooling was initiated and maintained according to current resuscitation guideline [18]. TH was initiated with infusion of cold saline and ice packages in axilla and groin to a target temperature of 33°C and hypothermia was maintained for 24 hours by a cooling dress. Passive rewarming was performed with a temperature increase of 0.5°C per hour.

### Statistical analysis

Statistical analyses were performed using GraphPad Prism® version 5. Continuous data was expressed as mean ± standard deviation (SD) when visual inspection suggested normally distribution. When not normally distributed, results were expressed as median and interquartile range. Categorical variables were reported as counts and percentages. Binary categorical variables were compared using Chi-squared test or Fisher’s exact test. Results were expressed as Odds Ratio (OR) and 95% Confidence Interval (CI). A P-value < 0.05 was considered statistical significant and all tests were two-sided.

## Results

The study population consisted of 173 patients over the age of 18. One patient was excluded from the study due to foreign nationality and therefore lack of fundamental information. Due to missing information in medical records; one patient had unknown initial rhythm, three patients had unknown time to ROSC and four patients had unknown discharge destination. These patients were still included in the study, although not in the analysis concerning their missing data. Finally, 172 patients were enrolled (Table 1). The vast majority of the patients admitted to the ICU after having CA with ROSC in the study period received TH treatment. Only 7 patients did not receive TH, they all suffered from severe instability due to cardiogenic shock and died within a few hours.

**Table 1:** **Baseline characteristics**

**Characteristics**	**Study group**
Gender - no./total no. (%)	
Male	131/172 (76)
Female	41/172 (24)
Age - year	
Mean ± SD	60.0 ± 12.9
≤60 years - no./total no. (%)	83 (48)
>60 years - no./total no. (%)	89 (52)
Mortality at discharge - no./total no. (%)	76/172 (44)
Mortality at 30 days from - CA no./total no. (%)	73/172 (42)
Mortality at 90 days from CA - no./total no. (%)	76/172 (44)
Location of cardiac arrest - no./total no. (%)	
Out-of-hospital	142/172 (83)
In hospital	30/172 (17)
VT/VF rhythm at presentation - no./total no.^1^ (%)	122/171 (71)
Non-VT/VF rhythm at presentation - no./total no. (%)	49/171 (29)
Asystole	41/171 (24)
Pulseless electrical activity (PEA)	4/171 (2.5)
Unknown non-shockable rhythm	4/171 (2.5)
Time from collapse to ROSC - no./total no.^2^ (%)	
<20 minutes	117/169 (69)
≥20 minutes	52/169 (31)
Etiology of cardiac arrest - no./total no. (%)	
Cardiac	124/172 (72)
Respiratory	28/172 (16)
Intoxication	7/172 (4)
Cerebral	3/172 (2)
Trauma	1/172 (0.5)
Malignant	1/172 (0.5)
Undefined	8/172 (5)
Discharge destination - no./total no.^3^ (%)	
Home/Rehabilitation facility	88/168 (52)
Nursing home/Death	80/168 (48)

CA: Cardiac arrest. VT: Ventricular tachycardia. VF: Ventricular fibrillation. ROSC: Return of Spontaneous Circulation.

^1^One patient data missing because of unknown initial rhythm.

^2^Three patients data missing because of unknown time to ROSC.

^3^Four patients data missing because of unknown discharge destination.

### Mortality

**Figure 1:**
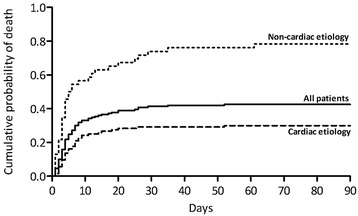
**90-day mortality and etiology.**

Among the entire patient population, there was an overall 90-day mortality rate of 44% (Figure 1). There was a significant difference in mortality rates among the entire patient population and patients with cardiac etiology only (P = 0.0262). Patients with a cardiac etiology had a 90-day mortality rate of 30%, compared with non-cardiac etiology; 78% (P < 0.0001). The 90-day mortality rate for all patients, regardless of etiology, presenting with VT/VF was the same as for patients with cardiac etiology (30%). For patients with cardiac etiology only, the mortality rate for VT/VF and non-VT/VF was 21% and 71% respectively (P < 0.0001).

**Figure 2:**
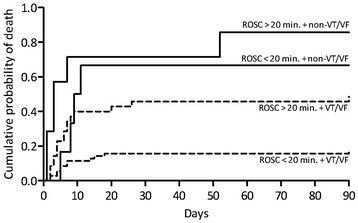
**90-day mortality, time to ROSC and initial rhythm for patients with cardiac etiology only.** ROSC = Return of spontaneous circulation. VT = Ventricular Tachycardia. VF = Ventricular Fibrillation.

Combining time to ROSC and initial rhythm revealed that the initial rhythm was the strongest predictor for survival. In Figure 2, the results of combining initial rhythm with time to ROSC among CA patients with a cardiac etiology can be seen. The mortality rate was 17% for VT/VF and ROSC < 20 min. and 49% for VT/VF and ROSC > 20 min. (P = 0.0004). It was 67% for non-VT/VF and ROSC < 20 min. and 86% for non-VT/VF and ROSC > 20 min. (P = 0.2362).

Survival was significantly better in younger patients. Mortality rate for patients with cardiac etiology and age ≤ or > 60 were 21% and 39% (P = 0.0325). Changing the age limit to ≤ and > 70, the difference in mortality was not significant, neither for all patients nor patients with cardiac etiology only.

OHCA patients had a trend towards a lower mortality than IHCA patients; 41% and 57% (P = 0.1055). There was a higher rate of cardiac etiology among patients with OHCA than IHCA, 80% and 37%.

### Neurological outcome

Among all 168 patients where neurological outcome could be evaluated, 52% had a favorable outcome (CPC 1–2). Only 10 patients (6%) were discharged to rehabilitation facility or nursing home and had thereby a poor neurologic outcome (CPC 3–4). Furthermore, 42% of the patients evaluated for neurological outcome died (CPC 5). 67% of patients with cardiac etiology had a favorable outcome, compared with 17% of patients with non-cardiac etiology (OR: 10.0; 95% CI, 4.28 to 23.4; P < 0.0001).

**Figure 3:**
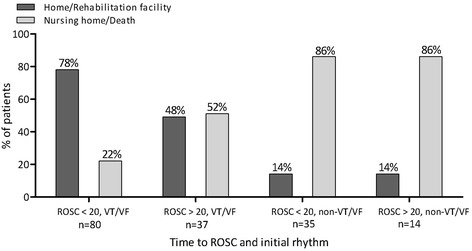
**Neurological outcome; time to ROSC and initial rhythm for patients with cardiac etiology.** ROSC = Return of spontaneous circulation. VT = Ventricular Tachycardia. VF = Ventricular Fibrillation.

The initial rhythm had a greater influence on the neurological outcome than time to ROSC. Patients with VT/VF had a significantly better outcome than patients with non-VT/VF, regardless of time to ROSC. The undeniably highest rate of favorable neurological outcome (83%) was seen among patients with cardiac etiology with VT/VF and ROSC < 20 min. In the same subgroup of patients, with ROSC > 20 min., the rate of favorable outcome was 51% (P < 0.0001). In patients with non-VT/VF and ROSC </> 20 min., the results were 33% and 14% respectively (Figure 3).

Age was not a predictor for a favorable neurological outcome (P = 0.1309).

### Initial rhythm related to other clinical factors

There was a considerably higher occurrence of cardiac etiology among patients with VT/VF, than among patients with non-VT/VF; 91% and 29%, respectively (OR: 25.2; 95% CI, 10.5 to 60.6; P < 0.0001). There was no significant difference in the distribution of time to ROSC </≥ 20 minutes between VT/VF and non-VT/VF rhythms (OR: 0.87; 95% CI, 0.42 to 1.80; P = 0.8543). Furthermore, there was no significant difference in the occurrence of VT/VF and non-VT/VF rhythms in neither age (P = 0.8665) nor gender (P = 0.1133).

Table 2 summarizes the predictors for survival and a favorable neurological outcome.

**Table 2:** **Analysis of clinical predictors for survival and favorable neurological outcome**

	**Survival**			**Favorable neurological outcome**		
	**OR**	**95% CI**	**P-value**	**OR**	**95% CI**	**P-value**
**VT/VF on presentation**	9.32	4.20 – 20.70	<0.0001	12.5	5.13 – 30.40	<0.0001
**Cardiac etiology**	8.60	3.89 – 19.00	<0.0001	10.0	4.28 – 23.40	<0.0001
**ROSC < 20 minutes**	2.45	1.26 – 4.78	0.0115	2.16	1.10 – 4.24	0.0287
**Age < 60 years**	1.89	1.03 – 3.48	0.0466	1.60	0.88 – 2.92	0.1309

## Discussion

In this retrospective cohort study, we investigated mortality, neurological outcome and possible predictors for favorable outcome in CA patients over a 3-year period at a Danish University Hospital. We investigated the importance of having a shockable initial rhythm versus a low time to ROSC for the mortality and neurological outcome. We found that an initial rhythm of VT/VF, CA of cardiac etiology, time to ROSC < 20 minutes and age < 60 years were all predictors for survival. The initial rhythm had a greater influence on the mortality and neurological outcome than time to ROSC.

### Mortality

The overall 90-day mortality was 44% and this is consistent with results of similar studies [12,19-21]. The majority of patients, who died, died within the first 20 days after their CA. This confirms that if the patients live through the first couple of weeks, they tend to survive. Also, patients who have a poor pre-CA health status and/or suffer from severe heart-/brain injury are likely to die as a direct result of the CA.

The 90-day mortality rate decreases significantly in patients with cardiac etiology compared with non-cardiac etiology, which illustrates that a cardiac cause is a predictor for good outcome. Our findings regarding a 90-day mortality rate of 30% are comparable to the results in the HACA study [9] and the study by Stub et al. [13]. The significant decrease in mortality in cardiac patients can be explained by a more frequent occurrence of VT/VF rhythms in these patients. We found that the odds of having a VT/VF rhythm of cardiac etiology are 25 times the odds of having a VT/VF rhythm of non-cardiac etiology. The better prognosis for patients with cardiac etiology may also be a result of more extended treatment options such as coronary artery catheterization, cardiac surgery, prophylactic Implantable Cardiac Defibrillator (ICD) therapy and pharmacological treatment.

Patients with VT/VF had a significantly lower mortality rate than patients with non-VT/VF and our results are consistent with the results of several other studies [5,22,23]. It is known that VT/VF has a better prognosis than non-VT/VF, as VT/VF can be defibrillated into a perfusing rhythm. Also, VT/VF is associated with a cardiac etiology.

### Neurological outcome

The fact that only 10 out of 168 patients were discharged to nursing home or rehabilitation facility indicates that patients, who survive, are likely to return to their previous neurological status. After the introduction of TH treatment, studies have shown that the number of CA patients discharged with a CPC score of 3–4 has decreased and correspondingly, the number of patients with CPC 1–2 has increased [8,20,22]. This may be a part of the explanation for the high number of patients being discharged with an intact neurological status after CA. Also, nowadays, in patients who are assessed as being at high risk of either dying or ending up in a vegetative state, futile treatment is often terminated.

We have demonstrated that the odds of being discharged with a favorable outcome are 10 times higher when having a cardiac etiology than a non-cardiac etiology. 67% of patients with a cardiac etiology had a favorable outcome, which is noticeably better than the findings by Bernard et al. [8], which was 49%. This is in spite of the fact that some of the patients in our study with cardiac etiology had non-VT/VF rhythms and Bernard et al. only included OHCA patients with VT/VF. One should though have in mind that the study by Bernard et al. was conducted over 10 years ago and treatment methods, especially regarding resuscitation, have been changed. First of all, in Denmark, the use of mobile intensive care units (ambulances with anesthesiologists and intensive care equipment) now allows for immediate initiation of professional CPR and subsequent cooling. Secondly, medical personnel are nowadays more experienced with TH treatment. Furthermore, our study population was significantly larger than in the study by Bernard et al. and the statistical certainty is therefore stronger.

Our results demonstrate that 91% of patients with a cardiac etiology present with VT/VF, which illustrates the strong correlation between these two variables. Our results regarding initial rhythm as a predictor for neurologic outcome are consistent with prior studies [12,13,19,24,25]. When only investigating the patients with a cardiac etiology, the odds of having a favorable neurological outcome when having a VT/VF rhythm are 8.6 times higher than the odds of favorable outcome and non-VT/VF. It is important, though, to be aware of the small number of patients with cardiac etiology and non-VT/VF, which may lead to a statistical uncertainty. Nevertheless, we can, based on our results, emphasize that a cardiac etiology and VT/VF have a strong positive impact on the outcome for CA patients.

### Time to ROSC associated with mortality and neurological outcome

We have illustrated that time from collapse to ROSC < 20 minutes is a positive predictor for survival and neurological outcome. Similar results have been shown in other studies [12,14,19]. When combining initial rhythm and time to ROSC, a remarkable finding is that the initial rhythm is the most important predictor for both survival and neurological outcome. In other words, the duration of non-sufficient circulation is not as important for survival and neurological outcome as the initial cardiac arrest rhythm. When having non-VT/VF, the time to ROSC is insignificant for both survival and neurological outcome in this study. Our results are verified by Herlitz et al. [22], who demonstrated that initial rhythm is the strongest predictor for survival, followed by time from call for and arrival of the ambulance, used as a marker for time to ROSC. However, there is a possibility that the initial rhythm itself can have an influence on time to ROSC, since a shockable rhythm can often be defibrillated into a perfusing rhythm. Our data describing mortality, initial rhythm and time to ROSC, show a trend towards a lower mortality among patients with cardiac etiology, but as the number of patients in each subgroup is small, it is difficult to draw any conclusions.

### Age associated with mortality and neurological outcome

The association between age and prognosis has been inconclusive in previous studies. There is a trend towards a lower mortality rate among younger patients [13,16,19,21,26] but there are also studies, which have not found an association between age and survival [12,27]. In our study, an age under 60 is a positive predictor for survival, regardless of etiology. Though, possible confounders should be considered, e.g. higher rate of comorbidities among elderly. The occurrence of VT/VF and non-VT/VF is not associated with age, neither is etiology.

Interestingly, changing the age intervals to </≥ 70 years, showed no statistical significance of lower mortality among patients < 70 years. Similar results are found in a Danish study, conducted by Larsen et al. [28]. The fact that the association between mortality and age </≥ 60 is statistically significant, suggests that the mortality rates are high in the group of patients between 60 and 70 years. Taking the above-mentioned results into consideration, it is indicated that active treatment of CA should not be excluded based on age.

### Location of CA associated with mortality and neurological outcome

Higher mortality rates among patients who suffered from IHCA compared with OHCA were also demonstrated in a study by Meaney et al. [24]. We assume that IHCA patients have a higher number of comorbidities and that they are more vulnerable on the actual time of the CA, which can lead to a selection bias in this study. The majority of IHCA patients had a non-cardiac etiology, mainly hypoxia, and therefore in higher risk of having a non-VT/VF rhythm and a worse prognosis. However, we have shown that 37% of the IHCA patients in this study suffered from CA of cardiac etiology. We included both ICHA and OHCA patients in this study, which of course may lead to a noticeable bias taken different etiologies into consideration. However, in our results, we analyze data from patients with cardiac and non-cardiac etiology separately, which narrows this bias considerably.

### Therapeutic hypothermia

The findings by Bernard et al. and Holzer et al. [8,9], have been further investigated and confirmed by other studies [12,13,19-21,25]. However, a recent International RCT by Nielsen et al. [29], reported no significant difference in outcome between OHCA patients with presumed cardiac cause, treated with hypothermia at a target temperature of 33°C versus 36°C, which led to the conclusion that the main consequence of TH may be preventing fever. Another recent Norwegian multicenter study by Lindner et al. [21], investigating prognostic factors for the use of TH in OHCA survivals, showed that after correcting for other prognostic factors, use of TH remained an independent predictor of improved survival. Hence, the discussion on whether or not TH is beneficial for outcome among CA patients is undoubtedly still on going.

TH does not appear to be associated with a higher number of patients ending up in a vegetative state. This may be influenced by the fact that the treatment of the patients at risk is terminated. Either way, the concerning patients are still treated with TH and are thereby given the chance of neurological improvement. However, our results are consistent with other studies, which report a high mortality and unfavorable neurological outcome for patients with non-VT/VF rhythms, regardless of treatment method [24,25,27].

### The future

In the past decade, the early treatment of CA victims has been improved in several ways, leading to more optimistic over all prognoses. A recent study by Wissenberg et al. [4], revealed that the bystander CPR rate in Denmark have increased significantly, from 21% in 2001 to 45% in 2010. Also, 30 day survival improved from 3,5% in 2001 to 10,8% in 2010. Although, because of co-occurrence of several initiatives to improve the management of OHCA patients in this period, the conclusion that the increased bystander rate is the main cause of the improved survival rate, cannot be drawn. On the other hand, increased bystander rate is highly correlated with the strong predictors of good outcome in this study; initial rhythm and time to ROSC.

### Limitations

There are several limitations to our study. We have conducted a retrospective cohort study and it is therefore subject to potential confounders and selection bias. Our data is retrospectively collected from medical records, leading to an information bias. Many of the investigated parameters are interpreted from clinical notes, which may have affected our results.

The patients comprise a selected group; all treated at a university hospital which has extended treatment options and personnel with extensive experience in managing patients with CA, indicating a selection bias. Also to be taken into consideration as a weakness, is the statistical uncertainty that occurs when several variables are compared, resulting in smaller study populations.

The strengths in our study are the relatively large patient population and the well-performed comparison between the initial rhythm and time to ROSC. We present strong statistical significance in well-defined tables.

## Conclusions

Our study results support the results of prior studies conducted on TH treated CA patients. 44% of all patients died and 52% of all patients were discharged with a favorable outcome. Predictors for survival and a favorable neurological outcome were VT/VF as presenting rhythm, CA of cardiac origin and time to ROSC < 20 minutes. Age < 60 years was a predictor for survival only.

There is a strong correlation between initial rhythm and etiology. Together and individually, these parameters are strong predictors for both survival and a favorable neurological outcome.

When combining initial rhythm and time to ROSC, we found that the duration of non-sufficient circulation during CA is not as important for survival and favorable neurological outcome as an initial rhythm of VT/VF.
